# Incidence and survival of hematological cancers among adults ages ≥75 years

**DOI:** 10.1002/cam4.1461

**Published:** 2018-04-13

**Authors:** Jessica L. Krok‐Schoen, James L. Fisher, Julie A. Stephens, Alice Mims, Sabarish Ayyappan, Jennifer A. Woyach, Ashley E. Rosko

**Affiliations:** ^1^ Division of Medical Dietetics and Health Sciences School of Health and Rehabilitation Sciences College of Medicine The Ohio State University Columbus Ohio; ^2^ Comprehensive Cancer Center The Ohio State University Columbus Ohio; ^3^ Arthur G. James Cancer Hospital and Richard J. Solove Research Institute Columbus Ohio; ^4^ Center for Biostatistics The Ohio State University Columbus Ohio; ^5^ Division of Hematology Department of Internal Medicine College of Medicine The Ohio State University Columbus Ohio

**Keywords:** Cancer, elderly, hematologic malignancies, older adults, SEER Program

## Abstract

Evaluating population‐based data of hematologic malignancies (HMs) in older adults provides prognostic information for this growing demographic. Incidence rates and one‐ and five‐year relative survival rates were examined for specific HMs among adults ages ≥75 years using data from the Surveillance, Epidemiology and End Results (SEER) Program. Hematologic malignancy cases (Hodgkin lymphoma (HL), non‐Hodgkin lymphoma (NHL), multiple myeloma (MM), acute lymphocytic leukemia (ALL), chronic lymphocytic leukemia (CLL), acute myeloid leukemia (AML), and chronic myeloid leukemia (CML)) were reported to one of 18 SEER registries. Recent average annual (2010–2014) incidence rates and incidence trends from 1973 to 2014 were examined for cases ages ≥75 years. One‐ and five‐year relative cancer survival rates were examined for adults ages ≥75 years diagnosed 2007–2013, with follow‐up into 2014. From 1973 to 2014, incidence rates increased for NHL, MM, and AML, decreased for HL, and remained relatively stable for ALL, CLL, and CML among adults ages ≥75 years. The highest one‐ and five‐year relative survival rates were observed among adults with CLL ages 75–84 years (1 year: 91.8% (95% CI = 91.8–90.8)) and 5 years: 76.5% (95% CI = 74.2–78.6)). The lowest one‐ and five‐year survival rates were observed among adults with AML ages 75–84 (1 year: 18.2% (95% CI = 74.2–78.6) and 5 years: 2.7% (95% CI = 2.0–3.6)). Survival for older adults ages ≥75 years with HMs is poor, particularly for acute leukemia. Understanding the heterogeneity in HM outcomes among older patients may help clinicians better address the hematological cancer burden and mortality in the aging population.

## Introduction

Hematological malignancies (HMs) are a group of blood cancers that vary in incidence, etiology, prognosis, and survival 1. HMs, broadly categorized into Hodgkin lymphoma (HL), non‐Hodgkin lymphoma (HL), multiple myeloma (MM), and leukemia, effect a disproportionate number of older adults resulting in significant morbidity and mortality. HMs collectively account for the fourth most common malignancy; 174,250 people will be diagnosed with HMs in 2018 and approximately 1.29 million people are living with HMs 2. Half of HMs are diagnosed in those 65 years and older and 70% of cancer deaths occur in this same population 3.

The U.S. population is aging and, by 2030, a 67% increase in overall cancer incidence is expected among the older adult population (65 years and older) 4. This growing demographic will have a profound impact on the incidence of HMs. Currently, the median age at diagnosis is 64 years for chronic myeloid leukemia (CML), 67 years for NHL, 68 years for acute myeloid leukemia (AML), 69 years for MM, and 70 years for chronic lymphocytic leukemia (CLL) 5, 6. ALL and HL have bimodal distributions, as they are found commonly both in those younger than 35 years (66.2% and 43.5% of new cases, respectively) and in older adults (11.7% and 17.9% of new cases, respectively) 5, 6. Survival probability for older adults with HMs is highly variable and dependent upon patient characteristics and treatment modalities. Prognoses for older adults diagnosed with HMs are often presented for only one age group (either all ages or 65 years and older), without respect to the potential high variation in prognosis within this relatively large age range; this categorization oversimplifies outcomes for aging adults, as prognoses for those 65–74 years are likely substantively different from those 85 years and older 7, 8, 9, 10, 11.

Limited research has examined potential differences between older age groups (e.g., 65–74 vs. 75–84 years) in improvements in HM incidence and survival 9, 12, 13. Thus, this study comprehensively examined incidence and survival for HMs among patient populations ages 75–84 years and 85 years and older. The study goal was to provide insight into cancer trends in the eldest older adult population that can be used to guide clinical decision making, provide prognostic information for patients, clinicians, researchers and public health practitioners, and identify gaps for future research.

## Methods

Data from the National Cancer Institute's Surveillance, Epidemiology and End Results (SEER) Program were used for these analyses. SEER is a collection of 18 high quality population‐based cancer registries with very high estimated completeness of reporting 14. These registries capture data covering approximately 30% of the U.S. population. All data were publicly available, de‐identified, and exempted from Institutional Review Board review.

Adults ages younger than 75 years, 75–84 years, and 85 years and older diagnosed with HL, NHL, MM, ALL, CLL, AML, and CML were included in analyses pertaining to incidence and relative survival rates. Years of diagnosis for incidence and relative survival rates were chosen because they are the most recent years available and they are consistent with years included in summary statistics reported in the Cancer Statistics Review 3.

Using SEER*Stat statistical software 15, cancer incidence rates were calculated for cases diagnosed from 2010 to 2014 and changes in incidence rates over time were examined for cases diagnosed from 1973 to 2014. For comparisons of 2010–2014 incidence data and the relative survival rates, 18 SEER registries were used 14. For comparisons of trends in incidence rates from 1973 to 2014, data from the original 9 SEER registries were used 16. Annual percentage changes (APC), using weighted least squares, and associated *P*‐values were calculated to determine whether incidence rates changed over time and whether any change is statistically significant. All cancer incidence rates were age‐adjusted using the 2000 U.S. standard population (19 age groups – Census P25‐1130).

One‐ and five‐year relative cancer survival rates for HL, NHL, MM, ALL, CLL, AML, and CML were examined for men and women ages 75 years and older, diagnosed from 2007 to 2013 and followed into 2014. One‐ and five‐year relative cancer survival rates were also calculated as a reference for men and women younger than 75 years. SEER describes relative survival as ‘a net survival measure representing cancer survival in the absence of other causes of death. Relative survival is defined as the ratio of the proportion of observed survivors in a cohort of cancer patients to the proportion of expected survivors in a comparable set of cancer free individuals. The formulation is based on the assumption of independent competing causes of death. The relative survival adjusts for the general survival of the U.S. population for that race, sex, age, and date at which the age was coded’. In addition, survival curves were created, using Kaplan‐Meier methods, for those ages younger than 75 years, 75–84 years, and 85 years and older for each of the HMs for overall survival. The SEER field ‘Survival Months’ was used as the overall survival follow‐up. It was defined as the time from diagnosis until the minimum of the date of last contact or study cutoff date for patients with vital status of ‘dead’ or ‘unknown’, or the study cutoff date (12/31/2013) for patients with a vital status of ‘alive’. Only cases for which there were complete dates were used to create survival curves. Stata software (version 14.2, StataCorp, College Station, TX) was used to create survival curves.

Supplementary sex‐specific analyses were conducted for all results and are presented in online Appendices [Link], [Link], [Link], [Link], [Link], [Link], [Link], [Link].

## Results

### Incidence

There were 55,927 men and women ages 75 years and older and diagnosed with HMs of interest (HL, NHL, MM, ALL, CLL, AML, and CML) in the 18 SEER registries from 2010 to 2014. Among adults ages 75–84 years, the age‐adjusted cancer incidence rates per 100,000 individuals were 4.4 for HL, 110.9 for NHL, 42.7 for MM, 1.9 for ALL, 32.1 for CLL, 25.4 for AML, and 8.9 CML from 2010 to 2014 (not shown in tables/figures). For adults ages 85 years and older, the age‐adjusted cancer incidence rates per 100,000 individuals were 3.5 for HL, 109.7 for NHL, 36.6 for MM, 1.5 for ALL, 36.9 for CLL, 26.5 for AML, and 10.3 CML from 2010 to 2014 (not shown in tables/figures).

**Figure 1:**
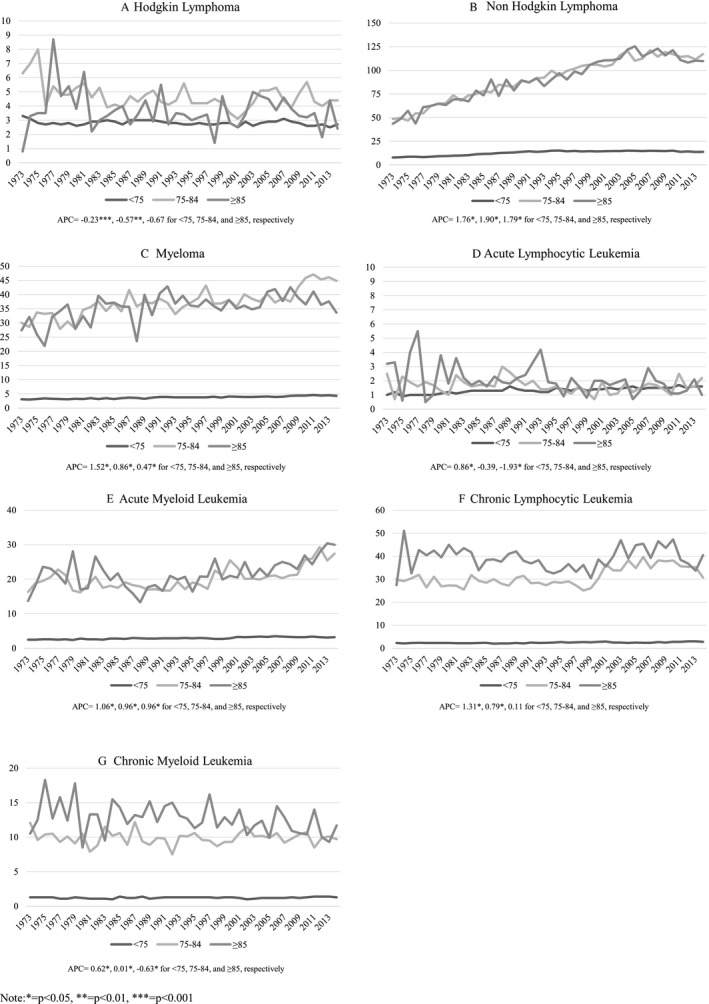
(A–G) Incidence of hematological cancers over time among adults ages <75, 75–84, and ≥85 from 1973 to 2014.

Figure 1A–G show the incidence trends of HL, NHL, MM, ALL, AML, CLL, CML among men and women ages 75–84 years, and 85 years and older from 1973 to 2014. Trends for men and women younger than 75 years are included as a reference. Incidence rates increased for NHL, MM, and AML, decreased for HL (excluding the non‐significant APC for the ≥85 group) and were relatively stable for ALL, CLL, and CML among adults ages 75–84 years and 85 years and older from 1973 to 2014. Overall, adults ages 75–84 years and 85 years and older had higher incidence rates of all HMs from 1973 to 2014, compared to those younger than 75 years. Supplementary sex‐specific results can be found in Appendices S1 and S2. In general, incidence rates over time showed that men in both age groups had higher HM incidence rates than women.

### Survival

**Figure 2:**
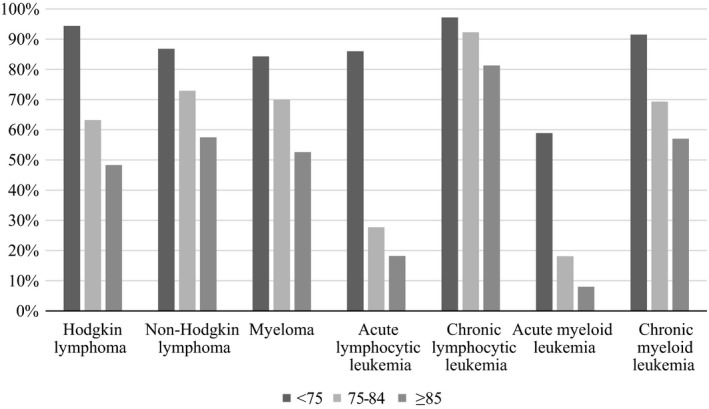
One‐year survival rates by disease type among adults ages <75, 75–84 and ≥85 diagnosed 2007–2013, with follow‐up into 2014.

Figure 2 shows one‐year relative survival rates by cancer type among those 75–84 years and 85 years and older, diagnosed 2007–2013. Trends for men and women younger than 75 years are included as a reference. The highest one‐year relative survival rate was observed among adults diagnosed with CLL (75–84 years: 91.8% (95% CI = 90.8–92.7; 85 years and older: 81.6% (95% CI = 79.5–83.6)). The lowest relative survival rates for both age groups was observed for AML which had one‐year survival rates of 18.2% (95% CI = 16.9–19.6) and 7.5% (95% CI = 6.1–9.0) for 75–84 and ≥85 year olds, respectively. The one‐year relative survival rates were consistently higher for adults ages 75–84 years compared those ages 85 years and older. The largest percentage difference was observed for NHL, with adults ages 75–84 years having a 72.4% (95% CI = 71.7–73.1) one‐year relative survival rate compared to adults ages 85 years and older having a 57.3% (95% CI = 56.0–58.6) one‐year relative survival rate. Supplementary sex‐specific results can be found in Appendices S3 and S4. Overall, the one‐year relative survival rates were similar for men and women.

**Figure 3:**
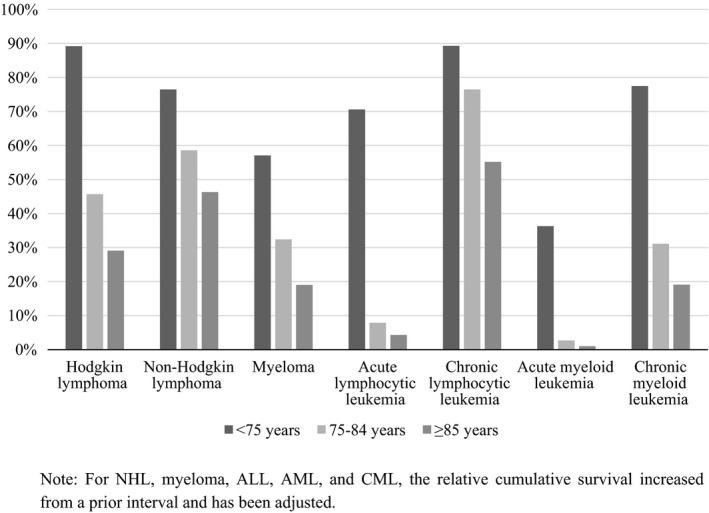
Five‐year survival rates by disease type among adults ages <75, 75–84 and ≥85 diagnosed 2007–2013, with follow‐up into 2014.

Figure 3 shows the five‐year relative survival rates by disease type among adults ages 75–84 and ≥85 diagnosed 2007–2013, with follow‐up into 2014. Trends for men and women younger than 75 years are included as a reference. As observed in one‐year relative survival rates, the highest five‐year relative survival rate, 76.5% (95% CI = 74.2–78.6) and 55.2% (95% CI = 50.3–59.8), was observed among adults ages 75–84 years and 85 years and older diagnosed with CLL, respectively. This was the largest survival difference (21.3%) between the two age groups. The lowest five‐year relative survival rates for both age groups were observed for AML, which had rates of 2.7% (95% CI = 2.0–3.6) and 1.0% (95% CI = 0.4–2.1) for 75–84 and ≥85 year olds, respectively. As observed in the one‐year relative survival rates, the five‐year relative survival rates were consistently higher for patients ages 75–84 years compared those ages 85 years and older. Supplementary sex‐specific results can be found in Appendices S5 and S6.

**Figure 4:**
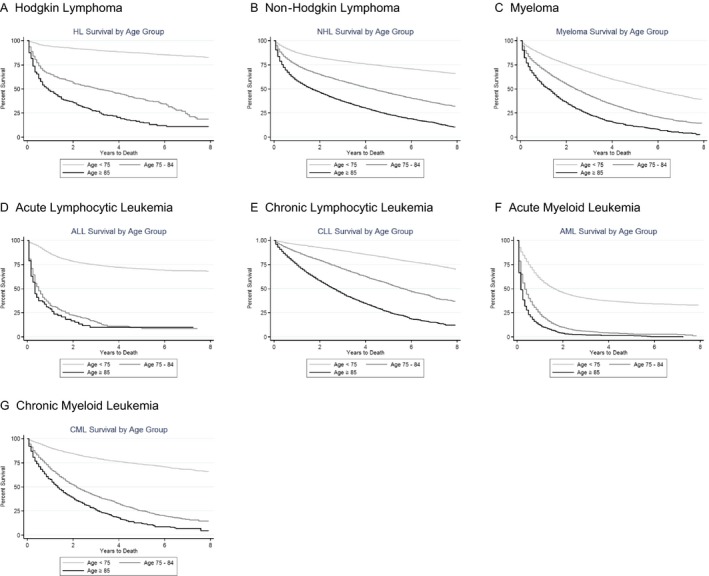
(A–G) Kaplan‐Meier survival curves of hematological cancers among adults ages <75, 75–84 and ≥85, diagnosed 2007–2013.

Figure 4A–G show Kaplan‐Meier survival curves for overall survival by cancer type among those younger than 75 years, 75–84 years, and 85 years and older, diagnosed 2007–2013 and followed through December 31, 2013. The survival curves show clear differences in overall survival among those younger than 75 years and those ages 75–84 years and 85 years and older for each HM. In addition, adults ages ≥85 had poorer overall survival than adults ages 75–84 years for HL, NHL, MM, CLL, and CML. The overall survival was similar for the two oldest age groups for AML and ALL. Supplementary sex‐specific results can be found in Appendices S7 and S8. Overall, the survival curves by sex were relatively similar; however, there were some notable differences for HL, NHL, CML, CLL, and ALL. Women ages <75 had the greatest survival for each HM, and men ages ≥85 had poorest survival for HL, NHL, CLL, and CML.

## Discussion

Current information about incidence and survival in older adult populations with HM is lacking. Understanding the heterogeneity in HM outcomes among older patients is warranted as it may help clinicians better address the hematological cancer burden and mortality in the aging population. This study, using population‐level data, found that one‐ and five‐year relative survival rates for older adults with AML and ALL is poor. It also demonstrated that older adult MM patients fare poorly, in contrast to their younger counterparts. In contrast, results indicated that older adults with HL and NHL are living longer.

Similarities and differences by sex regarding incidence and survival were found in this study. Incidence rate over time showed that men in both age groups had higher HM incidence rates than women. Incidence rates and one‐year and five‐year survival rates by sex followed similar trends. Survival curves did differ by sex regarding HL, NHL, CML, CLL, and ALL. These results indicate the need for clinicians to consider the age group and sex of their patients regarding treatment options and prognosis.

Older adults with HMs are a highly heterogeneous population in terms of health as some patients are highly functional and fit while others present cumulative deficits associated with geriatric syndromes and aging. In addition, a blood cancer diagnosis can significantly and abruptly change health status in the older adult. Equally heterogeneous are the treatment options available for older adults with HMs which are dependent on factors such as the disease, comorbidities, functional reserve, and perceived treatment tolerance. There is an emerging recognition that age itself is not the deciding factor for treatment allocation. A focus on physiological age, functional capacity, geriatric assessments, and calculators for chemotherapy toxicity is increasingly being recognized as tools to guide treatment decision making for the older adults with cancer 10, 17, 18.

One of the most recognized areas for clinical improvement is older adults with AML 19. Results indicated that the one‐year relative survival for AML is 18.2% and five‐year relative survival is 2.7% for adults ages 75–84 with half of all patients diagnosed with AML falling into this age bracket. In general, older patients with AML are categorized as ≥60 years of age and are known to have a more aggressive phenotype of AML, higher risk of multi‐drug resistance, and more frequent comorbidities than their younger counterparts 8, 20. Older AML patients are also more likely to have poor risk cytogenetic and molecular features with increased likelihood of secondary AML which also is known to lessen response to standard intensive chemotherapy 21, 22. Intensive induction chemotherapy risks have been shown to outweigh the benefits in patients over the age of 80 having approximately 40–50% early death rate 12, 23. Although allogeneic transplant is being offered more frequently in older AML patients, due to high risk of treatment‐related mortality, this is not standardly offered in patients ages ≥75 years. Therefore, potential treatment options for patients ages ≥75 years includes palliative chemotherapy (such as hypomethylating agents or low‐intensity cytarabine), supportive care, or potential treatment on a clinical trial. Although survival benefit has been shown for older patients with treatment versus best supportive care, 60% of older AML patients are not offered any therapy for their disease. Furthermore, clinical trial enrollment is infrequent with 78% of older adults with AML excluded from clinical trial research 24 although some individual centers report enrollment for adults ages >60 years as high as 21% 12, 25. In order to improve upon the survival rates and address the disparities in treatment options for older patients with AML, novel approaches targeting older patients are needed. One such approach is through The Leukemia and Lymphoma Society's Beat AML Master Trial which is a multi‐institutional trial for older (ages ≥60 years) newly diagnosed AML patients to perform upfront molecular sequencing which then in turn, determines a novel targeted therapeutic approach.

This study also found increasing rates of MM over time among those ages ≥75 years. Evaluating an aging population with MM is important as 35% of MM patients are diagnosed at ≥75 years, including 10% at ≥85 years 26. MM deaths overall are highest in patients ages ≥75 years, and early mortality is most common in those ≥70 years 27, 28. Similar to older adults with HM, survival disparities for older adults with MM is multi‐factorial and is secondary to treatment and transplant allocation differences, therapy toxicity, drug discontinuation, and individual patient physiologic reserve. Increasingly, MM therapy is becoming ‘personalized’ to improve tolerability, optimize efficacy, and ultimately survival. Future directions to improve MM outcomes in aging adults include improving transplant utilization, a sensitive disease response assessment, and optimal duration avoiding under or overtreatment.

Older adults with lymphoma present with similar clinical and prognostic characteristics, but have inferior outcomes in comparison with the younger population as found in this study 29, 30, 31, 32. Although there are differences in tumor biology across hematologic malignancies, under‐representation of older adults in clinical trials has hampered significant progress 33, 34, 35, 36. HL, a disease with bimodal distribution, is curable in the younger population; however, outcomes are not matched in the elderly with their inability to tolerate standard combination chemotherapy 37, 38. Recently, dedicated studies in older adults, using such agents as brentuximab, have found to be well tolerated and provide durable response as monotherapy in the frontline setting in older adults with HL 39, 40. Diffuse large B‐cell lymphoma is the most common type of NHL, and historically portends a poorer prognosis in older adults 31, 41, 42. These studies indicate improved survival over time, perhaps in part to, their focus on older adults with NHL, optimizing dose, and use of anthracyclines with NHL 43, 44, 45, 46.

This study found that older adults with CLL do relatively well; however, these study's data are limited as it does not differentiate between patients who require therapy at diagnosis compared to the larger group that is monitored without therapy at diagnosis. However, there was a large difference in relative survival rates between adults ages 75–84 and ≥85 years, suggesting that there may be disease‐related survival differences between these groups. This may reflect that standard of care chemoimmunotherapy can be more toxic in older patients or conversely, that older patients were not offered treatment at all. The recent introduction of oral targeted therapies in this disease is likely to alter the results observed here because of improved tolerability with these agents that may expand access to therapy to more medically fragile patients.

In summary, this study demonstrates increased incidence of NHL, MM, and AML over time, decreased incidence of HL, and stable rates for ALL, CLL, and CML for older adults ages ≥75 years. Results found that adults ages ≥75 years with NHL and CLL had the highest one‐ and five‐year relative survival rates approaching 50%. Older adults with MM and CML have a five‐year relative survival of approximately 30%. Older adults with AML and ALL have a very poor one‐ and five‐year relative survival rates. As the population ages, the incidence of HMs will most likely increase, warranting more research in this age group. One area of focus should be on treatment options due to the complexity of managing the disease along with other health factors related to aging. It is essential that clinical trials be designed to specifically test treatment regimens among older adults with HMs due to the lack of relevant and robust clinical trial data among this unique and growing patient population.

## Conflict of Interest

None of the authors have a conflict of interest.

## Supporting information


**Appendix S1 (a–g).** Incidence of hematological cancers over time among men aged <75, 75–84, and ≥85 from 1973 to 2014.

**Appendix S2 (a‐g)**. Incidence of hematological cancers over time among women aged <75, 75–84, and ≥85 from 1973 to 2014.

**Appendix S3**. 1‐year survival rates by disease type among men ages <75, 75–84, and ≥85 diagnosed 2007–2013, with follow‐up into 2014.

**Appendix S4**. 1‐year survival rates by disease type among women ages <75, 75–84, and ≥85 diagnosed 2007–2013, with follow‐up into 2014.

**Appendix S5**. Five‐year survival rates by disease type among men ages <75, 75–84, and ≥85 diagnosed 2007–2013, with follow‐up into 2014.

**Appendix S6**. Five‐year survival rates by disease type among women ages <75, 75–84, and ≥85 diagnosed 2007–2013, with follow‐up into 2014.

**Appendix S7: Figure 7(a–g).** Kaplan‐Meier survival curves of hematological cancers among men ages <75, 75–84 and ≥85, diagnosed 2007–2013.

**Appendix S8: Figure 8(a–g)**. Kaplan‐Meier survival curves of hematological cancers among women ages <75, 75–84 and ≥85, diagnosed 2007–2013.
