# Supplementary material for: Incidence and survival of hematological cancers among adults ages ≥75 years
Source: Cancer Med. 2018 Apr 13;7(7):3425–33. doi: 10.1002/cam4.1461 (PMC6051144; doi:10.1002/cam4.1461)
Supplement: Supplementary file 1 — Appendix S1 (a–g). Incidence of hematological cancers over time among men aged <75, 75–84, and ≥85 from 1973 to 2014. [file CAM4-7-3425-s001.docx]

Appendix 1a-g: Incidence of hematological cancers over time among men aged <75, 75-84, and ≥85 from 1973-2014.

APC=-0.38***, -0.75**, -0.62 for <75, 75-84, and ≥85

Note: APC=Annual Percentage Change; N/A=Statistic could not be calculated; *=p<0.05, **p<0.01, ***p<0.001
