# Supplementary material for: Incidence and survival of hematological cancers among adults ages ≥75 years
Source: Cancer Med. 2018 Apr 13;7(7):3425–33. doi: 10.1002/cam4.1461 (PMC6051144; doi:10.1002/cam4.1461)
Supplement: Supplementary file 2 — Appendix S2 (a‐g). Incidence of hematological cancers over time among women aged <75, 75–84, and ≥85 from 1973 to 2014. [file CAM4-7-3425-s002.docx]

Appendix 2a-g: Incidence of hematological cancers over time among women aged <75, 75-84, and ≥85 from 1973-2014.

APC=-0.03,-0.56, N/A for <75, 75-84, and ≥85, respectively

Note: APC=Annual Percentage Change; N/A=Statistic could not be calculated; *=p<0.05, **p<0.01, ***p<0.001
