# Supplementary material for: Incidence and survival of hematological cancers among adults ages ≥75 years
Source: Cancer Med. 2018 Apr 13;7(7):3425–33. doi: 10.1002/cam4.1461 (PMC6051144; doi:10.1002/cam4.1461)
Supplement: Supplementary file 5 — Appendix S5. Five‐year survival rates by disease type among men ages <75, 75–84, and ≥85 diagnosed 2007–2013, with follow‐up into 2014. [file CAM4-7-3425-s005.docx]

Appendix 5. Five-year survival rates by disease type among men ages <75, 75-84, and ≥85 diagnosed 2007-2013, with follow-up into 2014.

Note: For NHL, myeloma, ALL, AML, and CML, the relative cumulative survival increased from a prior interval and has been adjusted.
