# Supplementary material for: Incidence and survival of hematological cancers among adults ages ≥75 years
Source: Cancer Med. 2018 Apr 13;7(7):3425–33. doi: 10.1002/cam4.1461 (PMC6051144; doi:10.1002/cam4.1461)

Appendix 7. Figures 7a-g: Kaplan-Meier survival curves of hematological cancers among men ages <75, 75-84 and ≥85, diagnosed 2007-2013.

a. Hodgkin Lymphoma b. Non-Hodgkin Lymphoma c. Myeloma


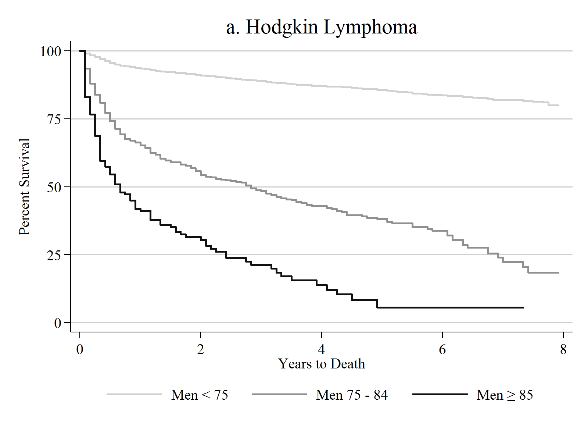

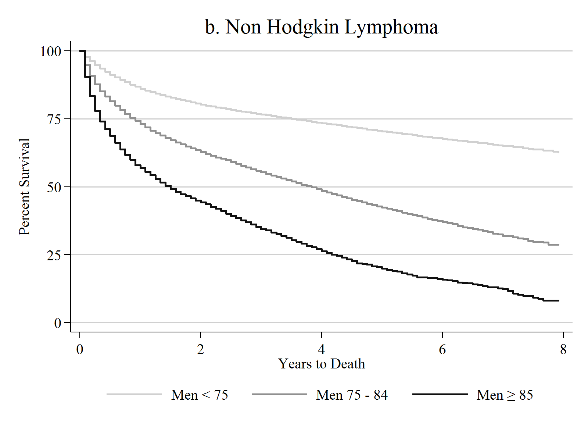

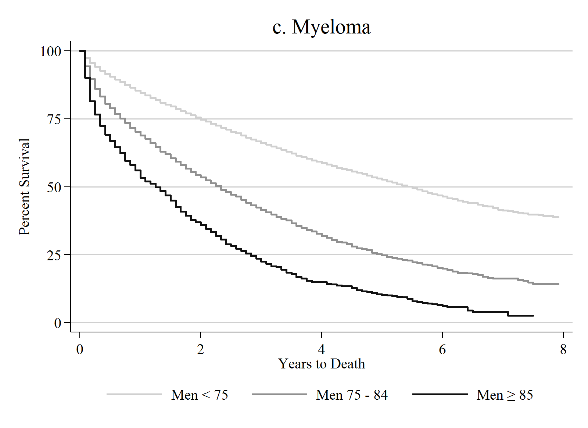


d. Acute Lymphocytic Leukemia e. Chronic Lymphocytic Leukemia f. Acute Myeloid Leukemia


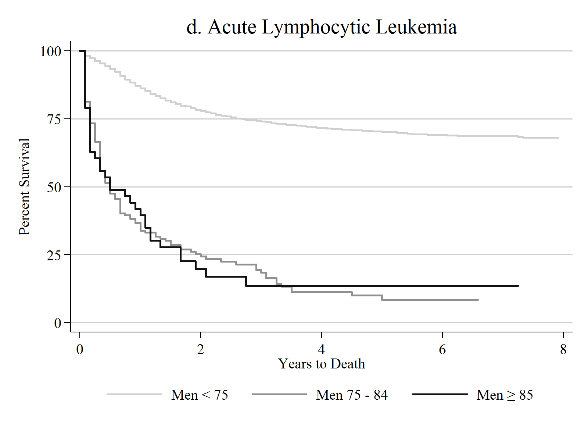

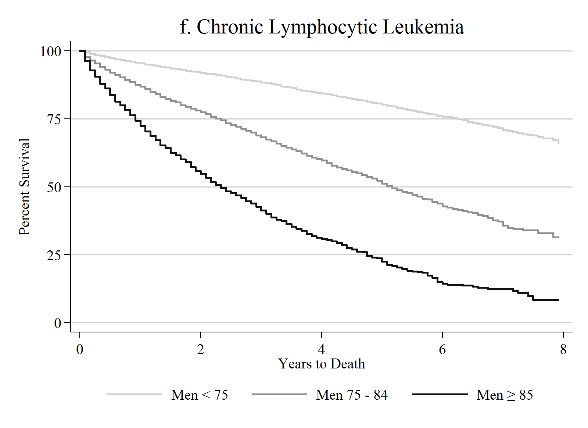

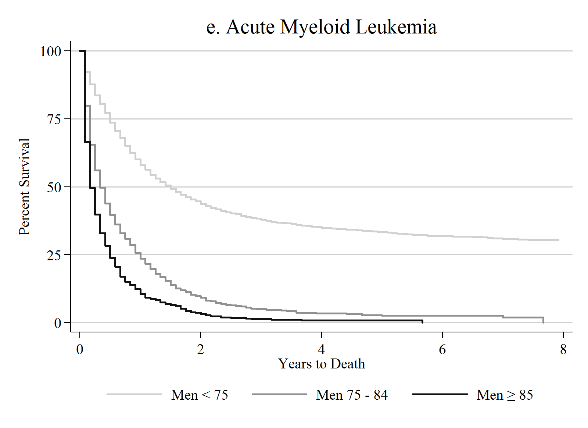


g. Chronic Myeloid Leukemia


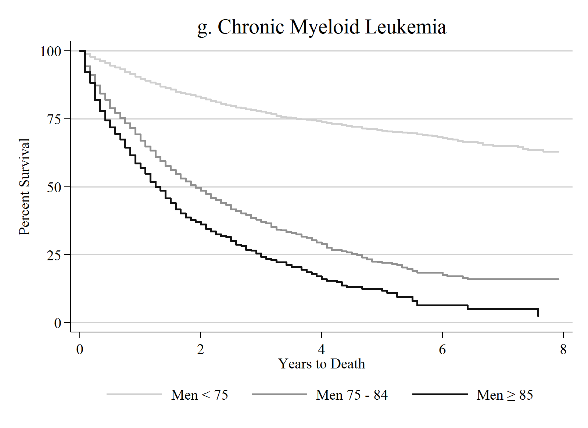

Supplement: Supplementary file 7 — Appendix S7: Figure 7(a–g). Kaplan‐Meier survival curves of hematological cancers among men ages <75, 75–84 and ≥85, diagnosed 2007–2013. [file CAM4-7-3425-s007.docx]
